# Addressing Antimicrobial Stewardship in Primary Care—Developing Patient Information Sheets Using Co-Design Methodology

**DOI:** 10.3390/antibiotics12030458

**Published:** 2023-02-24

**Authors:** Ruby Biezen, Stephen Ciavarella, Jo-Anne Manski-Nankervis, Tim Monaghan, Kirsty Buising

**Affiliations:** 1 Department of General Practice, The University of Melbourne, Melbourne, VIC 3004, Australia; 2 National Centre for Antimicrobial Stewardship, Department of Infectious Diseases, The University of Melbourne, Melbourne, VIC 3004, Australia; 3 The Guidance Group, Royal Melbourne Hospital, Melbourne, VIC 3000, Australia

**Keywords:** antibiotics, antimicrobial stewardship, co-design, consumers, general practice, inappropriate prescribing, patient information, primary care, primary care providers

## Abstract

Antibiotic resistance is a threat to global health, and inappropriate antibiotic use can be associated with adverse effects. Developing tools to encourage better communication between patients and general practitioners may reduce inappropriate use of antibiotics. The aim of the study was to develop shared decision support tools on common infections using a co-design methodology to address antimicrobial stewardship (AMS) in primary care. Three co-design/interview sessions were conducted with primary care providers and consumers between October 2019–April 2020 in Melbourne, Australia. Participants critiqued existing AMS tools, identified key elements required and optimised resulting prototypes. Primary care providers and consumers prioritised information to include in the AMS tools, such as when to see a doctor, management options, disease symptoms and cause of infection differently. However, both agreed content should be communicated in a plain, concise and logical manner, using inclusive and simple language accompanied by illustrations. Information sheets should be single-sided and A4-sized, appropriate for use before, during or after consultations. Co-design provided a collaborative forum to systematically design and develop products that meet the needs of both primary care providers and consumers. This resulted in the development of seven patient information sheets on common infections that encourage discussion of these infections, conservative management options and appropriate antibiotic use in primary care.

## 1. Introduction

Inappropriate antibiotic use is associated with increased antibiotic resistance, resulting in antibiotics no longer being effective, posing both immediate and long-term threats to human health [1,2]. Australia is one of the highest prescribers of antibiotics per capita, at double the rate of the lowest prescribers, such as Sweden and The Netherlands [3]. Most antibiotic prescribing in Australia occurs in primary care; it is, therefore, important that stewardship strategies are targeted for that setting.

Studies have indicated inappropriate prescribing of antibiotics may be due to many factors. These may include diagnostic uncertainty [4,5], physicians’ perceptions that patients would be more satisfied with the consultation if antibiotics were prescribed [6,7], physicians’ perceptions that patients expect antibiotics to be prescribed during consultations [8,9,10], and patients’ demands for antibiotics [11]. Recommendations from these studies included: encouraging better communication between patients and healthcare providers, using decision aids to assist shared decision-making between patients and healthcare providers, and delivering patient education on common infections.

The Clinical Care Standards in antimicrobial stewardship [12] specify that patients should expect to receive information about their clinical condition, its natural history and the treatment options available to them in a form that they can understand. Shared decision-making, where healthcare providers and patients discuss the benefits and risks of treatment options, can be used to guide healthcare decisions to help ensure clinical management meets patient expectations for health outcomes [13]. Patient decision aids have been developed for use in the areas of mental health, screening choices and treatment options, and antibiotic use [14,15,16,17,18,19]. They have been shown to be effective in reducing decisional conflicts [14], supporting patients with complicated treatment options [16], and encouraging shared decision-making [17]. However, many patient decision aids are not based on current evidence or have not been updated [20]. In addition, many are in clinical use despite limited studies to explore their effectiveness [21]. Therefore, a framework is needed to ensure new tools and resources for shared decision-making are developed with appropriate interpretation of evidence and adequate stakeholder review.

Currently, there are few patient decision aids for antibiotic use available in Australia [22]. Importantly, even fewer have been developed in consultation with both healthcare providers and patients using a documented, transparent process, nor have they been formally assessed for acceptability in the clinical setting. Therefore, the aim of this study was to co-design shared decision support tools with primary care providers (including general practitioners (GPs), a practice nurse, and a pharmacist) and consumers (members of the general public who attend a general practice at least once a year) to promote antimicrobial stewardship (AMS) in primary care.

## 2. Results

### 2.1. Participants

Data from the two co-design sessions and the participant interviews were collected between October 2019 to April 2020. Participants included five primary care providers (three GPs, a practice nurse and a pharmacist) and six consumers (an older male, an older female, a mother of a young child, a father of a teenager, a single male, and a 19-year-old male). Please see Table 1.

### 2.2. Co-Design Sessions and Interview Findings

**Figure 1:**
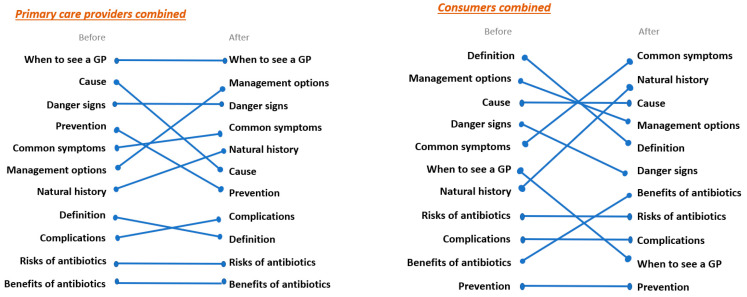
Ranking of 11 categories of information found in commonly used patient information sheets by primary care providers and consumers.

In the first co-design session, participants were asked to rank 11 categories of information found in commonly used patient information sheets (e.g., cause, symptoms, natural history of the illness, when to see a GP, prevention, risks of antibiotics) in order of importance before and after critiquing five patient information sheets commonly used in primary care. For primary care providers, ‘when to see a GP’ was considered the most important information to include in patient information sheets and the risks and benefits of antibiotics were considered the least important (Figure 1). The primary care provider ranking of the most and least important factors did not change after critiquing the five common patient information sheets. In contrast, consumers initially prioritised the definition and management options for their condition as the most important information. However, after critiquing the common information sheets, consumers ranked the most common symptoms and the natural history of the disease as the most important information to include. Information regarding prevention was considered the least important by consumers both before and after critiquing the common patient information sheets (Figure 1). This exercise demonstrated the differences between primary care providers and consumers in what they consider to be the most important information to include in patient information sheets.

Qualitative data from the two co-design sessions and subsequent participant interviews were analysed. Six major themes emerged: content, communication of content, design, delivery and access, usability, and engagement.

#### 2.2.1. Content

While critiquing common patient information sheets during the first co-design session, participants suggested that the most important content needed to be displayed in a format that was engaging for both primary care providers and patients.

*“We want the important stuff on the first page, and I think this stuff on the first page that’s irrelevant and meaningless should be on the back page and vice versa.”*

Participant 11, consumer, co-design session 1.

*“… the message it’s trying to convey, it’s a very simplified version of them writing it out and having lots of words and stats and things like that, so they’ve done it in a good way …”*

Participant 5, pharmacist, co-design session 1.

*“… and you don’t need the words ‘suprapubic pain’ … you just need to say if you’ve got blood in your urine or feel the need to pass urgently. I don’t think you need actual medical terms …”*

Participant 2, GP, co-design session 1.

*“… it’s easy to read, and it draws your attention to it with the pictures on the side, the decorations on the side, and it’s got the headings in bold, and short description. I think this is good for the public consumers.”*

Participant 8, consumer, co-design session 1.

Primary care providers often stated that information regarding ‘danger signs’ and ‘when to seek healthcare advice’ was important to manage medico-legal risks and to ensure patients were not dissuaded from seeking medical advice when needed.

*“If your symptoms aren’t improving, if you’re not getting better, and I think that’s why I personally prioritised that higher, because, that’s … if people aren’t getting better, not improving, to come back and see me.”*

Participant 1, GP, co-design session 2.

Having a generic warning in the shared decision support tools recommending that patients seek professional advice if they were unsure about whether they needed antibiotics was considered important, especially for at-risk patient groups, including those with chronic medical conditions.

Primary care providers all thought including information on bacterial versus viral pathogenesis of infections was too complex. Similarly, they reported that if antibiotics are not indicated for the condition addressed by shared decision support tools, there was little point in including information about the risks and benefits of antibiotics in that document.

*“With the benefits and risks, I just think it is a bit too complex to be on the person’s level of health literacy.”*

Participant 5, pharmacist, co-design session 1.

#### 2.2.2. Communication of Content

The use of simple, concise language suited to a Year 5 reading level (children aged between 10–11) was considered essential for communicating the necessary information. Avoiding complex medical terminology was also seen as particularly desirable.

It was considered important to use generic language where possible. However, it was acknowledged that confusion could arise because of the different brand names of medications patients may be familiar with.

*“I’ve had a patient who took their two Panadol, and they had their Panamax, and they had their paracetamol.”*

Participant 4, practice nurse, co-design session 2.

#### 2.2.3. Design

Bold font for headings and a balance between the use of words compared to pictures were considered significant design elements. Some design features became especially apparent when participants were asked to critique existing resources in co-design session 1, suggesting that important healthcare messages could otherwise be lost.

*“There’s nothing highlighted, there’s nothing to stand out, it’s all put with the same importance as everything else so—there’s no highlight of the fact when to see a doctor, it’s exactly the same text as symptoms.”*

Participant 10, consumer, co-design session 1 (reviewing existing patient information sheets).

Participants thought the information sheets needed to be engaging and that important content needed to be highlighted to make it easy for the patients to locate without having to search for relevant information.

*“I love the way you’ve bolded some of the things because I always either highlight or underline, I’m like, “These are the reasons I want you to come see me.”. So the fact that that’s already done would be good. I wouldn’t have to go searching for my highlighter …”*

Participant 1, GP, co-design session 2 (reviewing Shared Decision Support Patient Information Sheet Bronchitis prototype).

Both primary care providers and consumers considered it crucial that all the information was kept to a single-sided A4-sized page.

*“If it is going to be a two-pager, you’re not going to get them to turn over to read the second page.”*

Participant 5, pharmacist, co-design session 1.

*“If you’re going to get someone to do something, it needs to be really short.”*

Participant 1, GP, Session 3—Participant Interviews.

Including relevant pictures next to subheadings, along with the use of colour, was viewed as important for increasing readability. However, colour printing was deemed an expensive resource in a general practice environment; therefore, black and white copies were considered an acceptable alternative.

#### 2.2.4. Delivery and Access

Participants were encouraged to consider different delivery modes for the shared decision support tools. Having them available for patients to view in the community before the consultation was seen as an effective means of promoting the AMS message. Handouts, websites and online platforms were also seen as convenient modes of access to promote AMS both before and after GP consultations.

*“I was thinking—this is before they see the doctor. This might be at the chemist’s, or it could be online or in a childcare centre or a school, or I don’t know, whatever—a community thing where people see information.”*

Participant 2, GP, Session 3—Participant Interviews.

*“We could put it on the practice website, you know, in our resources section”*

Participant 1, GP, Session 3—Participant Interviews.

Participants agreed that handouts were the most appropriate form to promote AMS during a consultation. These handouts could be integrated into the practice management software and printed by a GP during a consultation or transferred to patients electronically.

*“I think this is really good … when I go and see him (GP) and quite often … he pulls up an information sheet, and he clicks ‘print’. Then he prints it and hands it to me as here’s some more information. So it would be nice if he has this in a file and can just hand it to me. Because when he actually hands it to me, hands me this information, I actually read it.”*

Participant 9, consumer, co-design session 2.

Using electronic delivery modes to distribute the shared decision support tools could give rise to barriers for patients without internet access or the ability to print the documents.

*“I also think quite a few, especially elderly patients, don’t use computers and emails. So I think for them, you need to have a written handout that their doctor gives them.”*

Participant 8, consumer, co-design session 1.

Participants also suggested that different modes of delivery would be required to ensure diverse groups within the community could access the information sheets. These might include translating these information sheets into different languages to accommodate culturally and linguistically diverse patients.

#### 2.2.5. Usability

The usability of the shared decision support tools was discussed at length during participant interviews. A key principle that emerged was that the tools needed to be readily accessible to facilitate use.

*“I use the three-click rule when we talk about all this co-design stuff. If they’re too far away … GPs won’t use it.”*

Participant 3, GP, Session 3—Participant Interviews.

Primary care providers identified that using the shared decision support tools might help save time during consultations for common infections and shorten the duration of such consultations.

*“Half the time—normally, I can make up my mind pretty quickly about what needs to happen … The hard part, then, is the … communication. It’s educating. If I’ve got a tool or resource, which is easily accessible—I know where it is, it sits on my screen, I can print it off, it saves me so much time.”*

Participant 1, GP, Session 3—Participant Interviews.

*“… I see so many people … who come in with symptoms suggestive of a viral sinusitis and often want to have that discussion about getting antibiotics. I think that the way the … tool is written and formatted would make it really … user-friendly and great to have it in a consult …”*

Participant 1, GP, Session 3—Participant Interviews.

#### 2.2.6. Engagement

During the first co-design meeting, participants suggested the shared decision support tools could make consumers aware of effective management options other than antibiotics. Furthermore, consumers might refer to the tools after the consultation had ended, potentially increasing their education about AMS.

*“[The tools] takes it away from the doctor and says, ‘What brings you here today?’, ‘That cough’s back again, I need the antibiotics.’ ‘Okay, let’s talk about that. There’s this new tool that I have …’”*.

Participant 3, GP, co-design session 1.

*“It’s another resource, then they’re going to walk away with … a piece of paper that says I’m not taking antibiotics, but I’m going to have some rest, sleep, drink more fluids”*.

Participant 5, pharmacist, co-design session 1.

Participants also said they were able to recognise non-antimicrobial management strategies for common infections by engaging with the shared decision support tools.

*“I think if I could give them this sheet with that and say, ‘Look, this is what the advice is saying. You don’t need antibiotics, you need to go home and take some Panadol and rest …”*.

Participant 1, GP, Session 3—Participant Interviews.

During the interviews, participants reported the draft patient information sheets contained sufficient information to allow consumers to decide whether they needed to see a primary care provider for the given condition and whether antibiotics may be needed.

*“I think if I have an ailment of some sort and I want to quickly decide before I contact a doctor what the best initial course of action is, I think these would be quite useful.”*.

Participant 10, consumer, Session 3—Participant Interviews.

At the end of the third co-design/participant interview phase, participants thought the shared decision support tools they helped co-design were easy to understand, concise, relevant, and consistent. Minor changes were suggested to wording, such as replacing ‘complex medical conditions’ with ‘other medical conditions’.

The outcome of this study was the development of seven shared decision support tools in the form of patient information sheets: acute bronchitis, middle ear infection, nose & sinus infection, sore throat, urinary tract infection, cellulitis and leg ulcers.

## 3. Discussion

The focus of this study was to develop robust shared decision support tools using a co-design methodology to promote appropriate antibiotic use in primary care. The co-design methodology provided a collaborative forum with a diverse group of participants to identify their key priorities and needs. This methodology allowed us to systematically design and develop a product that is acceptable and meets the needs of both primary care providers and patients. Together participants advised on the attributes of the shared decision support tools required to address their specific needs, including attributes related to the content, design, usability, communication, and access and delivery of the tools.

Both primary care providers and consumers emphasised the importance of communication using simple, concise and inclusive language. Design elements such as layout and formatting, with a balance between the use of words and pictures, were required to engage users and improve usability. Information needed to be unambiguous and relevant to the patient’s condition. Complex diagrams and numbers were not favoured as they were confusing to some. Tools such as patient information sheets and decision aids that contain relevant information can therefore assist patients in the decision process [13,23,24].

Participants also provided insights into suitable delivery modes and access points for using the shared decision support tools. Depending on the condition addressed and the availability of health care access, participants wanted these tools to be available before, during and after a GP consultation to improve patient education regarding common infections and to support them in deciding when to see their GP. This was particularly important because patients would often present to pharmacists for advice if they could not secure a GP appointment. It would therefore be imperative that the shared decision support tools are made available to all primary care providers to ensure uniform messaging to patients and to avoid patient confusion and misinterpretation.

The results from the co-design sessions showed that primary care providers and consumers prioritised what information is needed in the shared decision support tools differently. GPs and patients often have dissonant views when it comes to assessing and managing health outcomes [25,26,27]. In our study, consumers preferred to know more about common symptoms and the natural history of a condition rather than when to see a GP. Previous studies have also shown that primary care providers and patients have conflicting views, especially when it comes to antibiotic prescribing and use [27]. GPs sometimes perceive patients wanting a diagnosis, along with the reassurance that they are not severely ill, as pressure to prescribe antibiotics [4,27,28,29]. This suggests that communication is crucial to align primary care providers’ and patients’ expectations regarding appropriate antibiotic prescribing and use.

It is interesting to note that while common patient information sheets often included risks and benefits of antibiotics, these were considered as least important by our participants. This exercise demonstrated how important a co-design process (which involves end-users in the development process) is when developing any shared decision support tool.

Participants overwhelmingly wanted information sheets that included information about disease symptoms, signs to look out for, prevention, management and treatment strategies, and when to see a GP. While the initial aim of the study was for the shared decision support tools to only include categories of information found in common information sheets, which were prioritised as most important in the first co-design session, through the co-design process, we were able to include all 11 categories in the final product for the seven information sheets (Please see Files S1 and S2). These information sheets can therefore be used not only to encourage communication between primary care providers and patients but also to provide patients with a useful educational resource to refer to before or after a GP consultation [30,31].

As far as we are aware, this is one of the first studies that applied a co-design methodology to systematically develop shared decision support tools for AMS in primary care. Enabling both primary care providers and consumers to work directly with each other to develop seven patient information sheets was one of the strengths of this study. Instead of a traditional development of a tool/product designed by content experts, we gain an understanding of different participant perspectives, supporting the development of a more robust product. In addition, because the shared decision support tools were cross-referenced with Therapeutic Guidelines (Australia’s nationally endorsed guidelines), the tools are an evidence-based product.

The COVID-19 pandemic brought research-specific challenges to this study. We were unable to complete the third face-to-face co-design session as scheduled, but we were able to conduct one-on-one interviews with each of the participants. These interviews provided participants with the opportunity to privately share their perspectives rather than in a group environment. The feedback from the co-design sessions and the interviews were found to be consistent across all participants, hence validating the reliability of the participant responses. The strong relationships built with research participants throughout the study, as well as the collective belief in the value and importance of reducing inappropriate antibiotic use in the community, allowed us flexibility to adapt our study in light of these challenges. The resulting product is robust, evidence-based, and has the potential to reduce inappropriate antibiotic prescribing and use in primary care.

## 4. Materials and Methods

### 4.1. Study Design

This co-design study was designed to consist of three co-design sessions with primary care providers and consumers. Co-design sessions 1 and 2 were facilitated by the lead researcher (RB) with assistance from the medical student (SC), a GP (J-AM-N) and an infectious disease physician (KB). Due to the COVID-19 pandemic, the third co-design session was replaced by individual participant interviews.

### 4.2. Participant Recruitment

Participants consisted of three GPs, a practice nurse, a pharmacist, and six patients/consumers that attend a general practice for the majority of their medical care. They were recruited via the research team’s professional network, such as the National Centre for Antimicrobial Stewardship and the Victorian primary care practice-based Research and Education Network (VicREN) [32]. An advertisement for the project, a plain language statement and a consent form were sent to potential participants via email. Interested participants contacted the research team, who explained the project in detail and answered any questions. Consumers were recruited via the research team’s professional network, including consumer groups at the University. A diversity of consumers were recruited based on their age (older and younger participants) and family status (single, married and/or with children). All consent forms were signed by participants before commencement at each of the three sessions.

### 4.3. Data Collection and Analysis

#### 4.3.1. Co-Design Session 1

The first co-design session focused on what information participants considered important to include in patient decision aids/information sheets and by what mode(s) this information should be delivered. Using bronchitis as an example, participants were asked to rank 11 categories of information (common classifications in patient information sheets, e.g., cause, symptoms, natural illness, when to see a GP, prevention, risk of antibiotics) from most important to least important. Participants were then asked to critique five common patient information sheets, discussing what they liked and disliked about those information sheets. Participants were then asked to rank the 11 categories of information again in order of importance. Following this, participants provided feedback on how to optimise the information and delivery of the information in those materials. The session was recorded on video and audio.

For category rankings, each category was given a number from 1 to 11. The numbers were added together and grouped according to each category, for each participant group (primary care providers or consumers) as ‘before’ and ‘after’ critiquing the five common patient information sheets. Differences were recorded and compared. A positive difference showed an increase, and a negative difference showed a decrease from ‘before’ to ‘after’. Results were analysed descriptively in Excel. The audio of the co-design session was transcribed verbatim, any identifying information was removed, and common themes were identified using thematic analysis.

Following data analysis of the first co-design meeting, the research team drafted content for the shared decision support tools, which aligned with Therapeutic Guidelines [33]. This content was reviewed by a content expert group consisting of two GPs, one pharmacist, one infectious diseases physician, one aged care nurse and two microbiologists. The agreed draft content for a shared decision support tool focusing on bronchitis was provided to a graphic designer to develop the first prototype tool.

#### 4.3.2. Co-Design Session 2

The second-co-design session focused on the content, format, and layout of the bronchitis prototype of the shared decision support tool. The content was also discussed for six other conditions (tonsillitis, otitis media, rhinosinusitis, urinary tract infection, leg ulcers and cellulitis). These conditions were selected as they were the most common conditions resulting in antibiotics being prescribed in primary care conducted from a previous study [34]. The session was recorded on video and audio. The audio was transcribed verbatim, any identifying information was removed, and common themes were again identified using thematic analysis.

Feedback from the second co-design session was reviewed by the research team. The graphic designer refined the bronchitis information sheet based on the feedback received and applied the resulting formatting to information sheets for the other 6 conditions.

#### 4.3.3. Session 3/Participant Interviews

The third co-design meeting was scheduled for March 2020. However, due to COVID-19 restrictions enforced in Melbourne, Australia, it was replaced with individual semi-structured interviews that were conducted by the Zoom online platform or via telephone. In these interviews, conducted by RB and/or SC, participants were asked to provide feedback on the information sheets and how these tools could be delivered and used. Questions for primary care providers included: ‘Can you explain how you would use the shared decision support tool in your work? Can you provide an example?’ and ‘What form(s) do you think would be appropriate for use before/during/after a consultation with a patient’? Similarly, for consumers, questions included: ‘in what circumstances do you think the shared decision support tools will be helpful to you?’ These interviews were recorded and subsequently transcribed and analysed thematically. Drafts of the seven patient information sheets were also mailed to all participants with a request to provide written comments on the layout and content of the drafts. These were returned to the researcher via reply paid Express Post. Marked-up information sheets were summarised, and changes were provided to the graphic designer for refinement.

Qualitative data were analysed using a deductive thematic analysis approach using NVivo 12 (QSR International, Doncaster, Australia). The coding scheme was developed by grouping recurrent ideas during data analysis and refined under themes and subthemes. Data were coded independently by two researchers (SC and TM), with differences in perspective negotiated with a third researcher (RB) until a consensus was reached. The coding structure was discussed with the research team and refined until full agreement was attained. Overlapping themes were discussed as to how best to describe these themes to avoid repetitiveness. The thematic coding scheme adopted by the coders was described under six headings: content, communication of content, design, delivery and access, usability, and engagement.

Ethics approval was obtained from The University of Melbourne General Practice Human Ethics Advisory Group (Ethics ID: 1954925.1).

## 5. Conclusions

This study demonstrated that primary care providers and consumers have different priorities when it comes to what information is important for them to include in shared decision support tools. Our results indicated the importance of a co-design process involving end-users in the development when creating any such tool. Using this process, primary care providers and consumers were able to come to a mutual agreement on the content, format and layout, delivery mode and access of these shared decision support tools. This resulted in an end product that was simple, clear, and attractive to both primary care providers and patients to use. These patient information sheets summarised disease symptoms and signs, prevention, management and treatment strategies, and when to see a GP. The next step is to pilot these tools to assess their usability and acceptability across general practices in Victoria, Australia.

## Figures and Tables

**Table 1:** Demographics of primary care providers and consumers.

Primary Care Providers	Gender	Age (Years)	Years of Experience (Primary Care)
Participant 1 (GP)	Female	26–35	2
Participant 2 (GP)	Male	46–55	27
Participant 3 (GP)	Female	56–65	30
Participant 4 (Practice Nurse)	Female	26–35	5
Participant 5 (Pharmacist)	Female	26–35	1
**Consumers**	**Gender**	**Age (Years)**	**Number of GP Visits per Year**
Participant 6	Male	18–25	2
Participant 7	Male	>75	3
Participant 8	Female	66–75	4
Participant 9	Male	46–55	3
Participant 10	Female	36–45	3
Participant 11	Male	46–55	2

## Data Availability

Data can be available upon request.

## Supplementary Materials

The following supporting information can be downloaded at: https://www.mdpi.com/article/10.3390/antibiotics12030458/s1. File S1: Bronchitis patient information after Co-design session 1; File S2: Bronchitis patient information after Co-design session 3.
