# Supplementary material for: Addressing Antimicrobial Stewardship in Primary Care—Developing Patient Information Sheets Using Co-Design Methodology
Source: Antibiotics (Basel). 2023 Feb 24;12(3):458. doi: 10.3390/antibiotics12030458 (PMC10044618; doi:10.3390/antibiotics12030458)
Supplement: Supplementary file 1 [file antibiotics-12-00458-s001.zip › File S1 Bronchitis patient information after Co-design session 1.pdf]

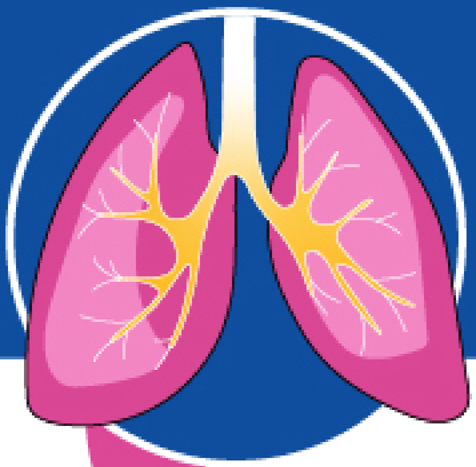

ACUTE BRONCHITIS

Acute Bronchitis is an inflammation of the airways in your lungs, most commonly caused by a **viral infection**.

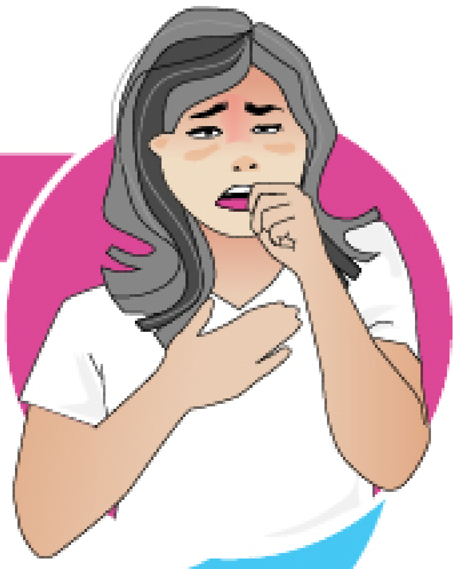

How does it make you feel?

You **cough a lot** and there may be **phlegm** in your cough. You may hear a **wheezing sound** when you breathe. Your cough might **wake you at night**. You may also have a **blocked nose**, **sore throat**, **mild headache** or **fever.**

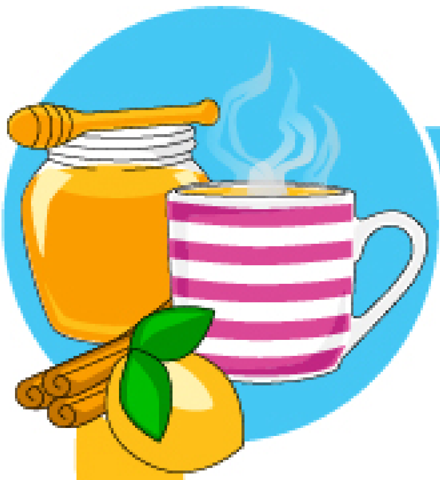

What can you do to feel better?

Light pain releif such as **Panadol** and **Nurofen** might ease your sore throat or mild headache, while simple remedies such as **warm drinks**, **honey** or **cough lozenges** may soothe your throat and ease coughing. Some people find **inhaling warm air** is helpful. Try to *avoid anything that irritates the airways*, such as cigarette smoke.

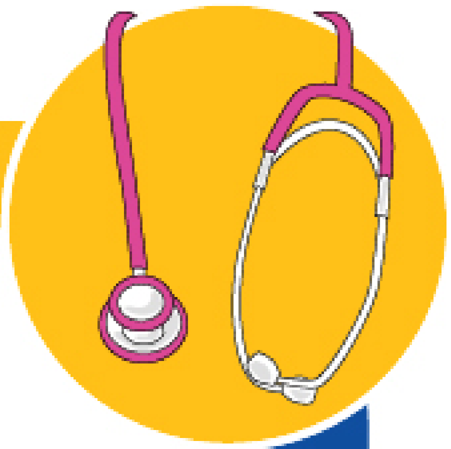

Do you need to see a doctor?

Usually bronchitis can be managed without the need for a doctor, and the cough normally goes away after 2-3 weeks. See your doctor if your symptoms take longer than 3 weeks, you experience **shortness of breath, confusion**, **disorientation**, **shaking** or **chills**, or if feel that you are getting worse or are worried.

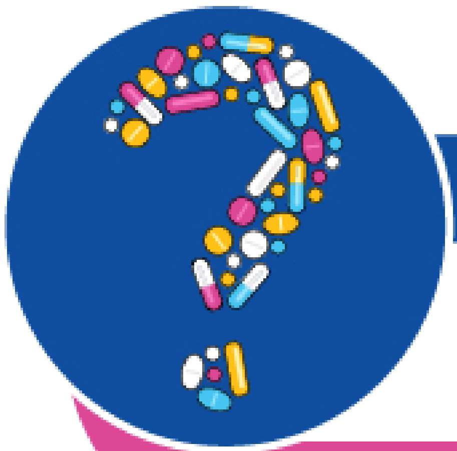

Will antibiotics help?

Not usually. Antibiotics can't kill viruses like the ones that cause bronchitis. They are made to kill bacterial infections.

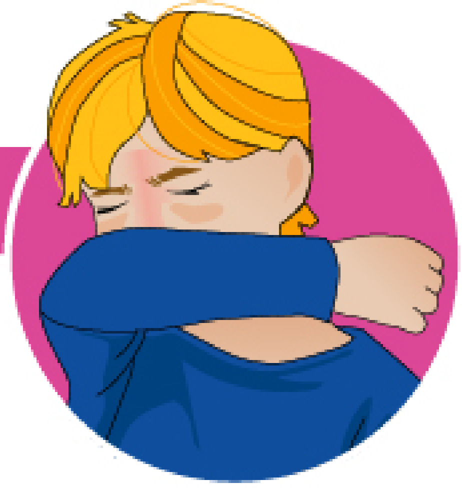

How can I stop it spreading?

The viruses that cause bronchitis can be spread to others through droplets from your nose or mouth. To minimize this, **cover your mouth with your elbow when you cough**. **Wash your hands** if you have coughed into them, especially before touching other people. Dispose of tissues after use, and try to **stay away from crowded places** where you might risk exposing other people.

NCAS
