# Supplementary material for: Addressing Antimicrobial Stewardship in Primary Care—Developing Patient Information Sheets Using Co-Design Methodology
Source: Antibiotics (Basel). 2023 Feb 24;12(3):458. doi: 10.3390/antibiotics12030458 (PMC10044618; doi:10.3390/antibiotics12030458)
Supplement: Supplementary file 1 [file antibiotics-12-00458-s001.zip › File S2 Bronchitis patient information after Co-design session 3.pdf]

ACUTE BRONCHITIS

NCAS
National Centre for Antimicrobial Stewardship

Bronchitis is an infection of the airways in the lungs, most commonly caused by a virus.

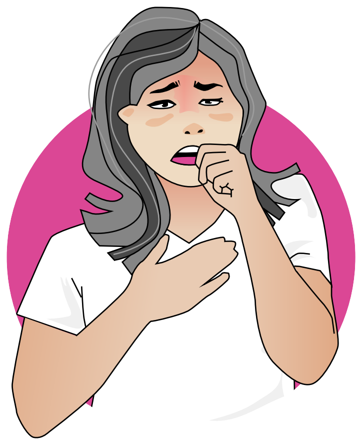

What does it feel like?

You will have a **cough** which may be associated with clear, yellow or green phlegm, **noisy breathing**, **blocked nose**, **sore throat**, **mild headache**, and **fever**.

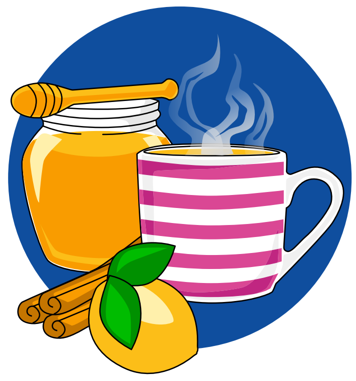

What can I do to feel better?

Bronchitis usually gets better on its own. **Paracetamol** and **ibuprofen**, **warm drinks**, **honey**, **cough lozenges** and **inhaling steam** from the shower may help ease your symptoms. Avoid anything that irritates the airways, such as cigarette smoke.

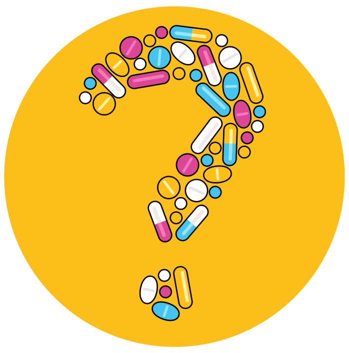

Will antibiotics help?

Antibiotics are not usually needed. Taking antibiotics when you don't need them can reduce how well they work if you do need them in the future.

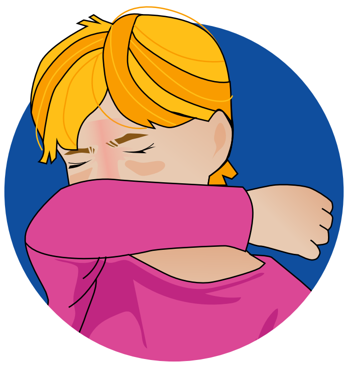

What can I do to stop it spreading?

Infections can spread to others when you cough, sneeze or blow your nose. **Cover your mouth with your elbow when you cough or sneeze**, **wash your hands** regularly, **dispose of tissues after use** and **stay away from crowded places while unwell**.

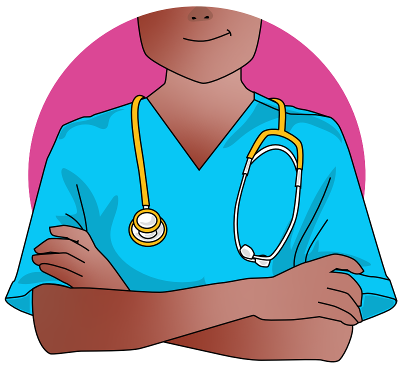

Do I need to see a doctor?

Not usually. The cough normally takes 2 to 3 weeks to go away. If your symptoms last longer or if you have **trouble breathing**, you are **feeling worse**, you have **other medical conditions such as emphysema**, or you are concerned, see your doctor.

The information in this factsheet is not intended to be a comprehensive guide. It is recommended to seek advice from a registered health care professional for diagnosis and answers to medical questions and to determine whether the recommendations are suitable for your circumstances.

© 2020 National Centre for Antimicrobial Stewardship

THE UNIVERSITY OF MELBOURNE
